# Prevalence, trends and associated factors of hypertension and diabetes mellitus in Bangladesh: Evidence from BHDS 2011 and 2017–18

**DOI:** 10.1371/journal.pone.0267243

**Published:** 2022-05-03

**Authors:** Nusrat Jahan Sathi, Md. Akhtarul Islam, Md. Sabbir Ahmed, Sheikh Mohammed Shariful Islam

**Affiliations:** 1 Statistics Discipline, Science, Engineering and Technology School, Khulna University, Khulna, Bangladesh; 2 Department of Community Health and Hygiene, Patuakhali Science and Technology University, Dumki, Patuakhali, Bangladesh; 3 Institute for Physical Activity and Nutrition, Deakin University, Melbourne, Victoria, Australia; University of Southern Queensland, AUSTRALIA

## Abstract

The evolving pandemic of non-communicable diseases like hypertension, diabetes mellitus are globally on the rise, and the trend is also escalating in Bangladesh. We aimed to assess the prevalence trend and associated factors of hypertension (HTN), diabetes mellitus (DM), and hypertension- diabetes mellitus combined (HDC) among Bangladeshi adults from 2011 to 2018. Two nationally representative cross-sectional data from Bangladesh Demographic and Health Survey (BDHS): 2011 and 2017–18 were utilized. According to baseline characteristics, the average annual rate of change (AARC) was applied to quantify the annual rate of increase/decrease in HTN, DM, and HDC from 2011 to 2018. The prevalence ratios of HTN, DM, and HDC were assessed through modified Poisson regression with robust error variance (PR, 95% Confidence Interval (CI)). The data were prepared in SPSS version 23 and exported to Stata version 13 for further analysis. Among 11,686 participants, the overall mean age of the study participants was 52.79 years, Standard Deviation (SD)±12.99, and 42.28% were female. From 2011–2018, HTN, DM, and HDC prevalence in Bangladesh has increased by 13, 3.2, and 3.1 percentage points, respectively. The average annual rate of increase was observed in the HTN and HDC prevalence by all socio-economic and demographic categories during 2011–2018. The prevalence of HDC among Chittagong residents was approximately double in 2018: 3.95% (2011) versus 6.59% (2018). Increased age, inactive workers, overweight adults, and adults in wealthy families were common risk factors associated with HTN, DM, and HDC in Bangladesh. The prevalence of developing HTN and HDC was significantly higher among adults aged ≥ 70 years (PR: 2.70, 95% CI: 2.42–3.00; PR: 2.97, 95% CI: 2.08–4.24, respectively). A comprehensive approach of different stakeholders is required to develop appropriate strategies, including appropriate weight management, adequate physical activity, and healthier food habits. Health agencies should take initiatives to spread awareness among people at an early age, but special attention is needed for older people and those at risk for NCDs.

## Introduction

Non-communicable diseases (NCDs) are long-term non-infectious diseases caused by different factors like genetic, socio-demographic, physical, biochemical, and behavioral [1]. According to the World Health Organization (WHO), NCDs are responsible for 70% of all deaths worldwide, of which more than 75% of deaths took place in low and middle-income countries (LMICs) [2]. The WHO predicted that deaths due to NCDs would be 52 million by 2030 [3]. Hypertension (HTN) and Diabetes Mellitus (DM) are the two most common and deadly NCDs worldwide, including stroke, kidney failure, disability, and millions of premature deaths [4]. Although HTN was considered a health concern for developed countries, recent data suggest that the prevalence is rapidly increasing in LMICs [5, 6]. More than 35% of adults from the South-Asian region suffer from HTN [5, 6]. Besides, the worldwide prevalence of DM among adults may rise to 10.2% by 2030 and 10.9% by 2045 [7].

In recent years, Bangladesh has been facing an epidemiological shift from communicable diseases to NCDs [8]. As a developing country, NCDs emerged as the leading cause of mortality and morbidity and became a crucial public health problem for Bangladesh [9]. Rapid urbanization, sedentary lifestyle, poor dietary habits, increased tobacco consumption, CVD rates may cause HTN and DM, rising in Bangladesh [4]. According to the WHO-NCDs progress monitor report, about 67% of all deaths in Bangladesh occurred due to NCDs [2]. In Bangladesh, nationwide HTN and DM data usually come through Bangladesh Demographic and Health Survey (BDHS). According to the most recent BDHS 2017–18, the prevalence of HTN among women and men aged 35+ were 45% and 34%, respectively [10]. On the other hand, the prevalence of DM was reported 14% for both men and women [10]. A recent systematic review and meta-analysis showed a conjoined prevalence of DM (7.8%) among the general population in Bangladesh [11]. Literature also suggests substantial regional inequalities in the prevalence of HTN (10% to 35%) and DM (6% to 19%) across Bangladesh [4].

Previously, efforts have been made to identify the factors associated with HTN and DM among Bangladeshi adults. Existing evidence reported higher age, higher educational attainment, and better wealth status as significant factors of HTN and DM among Bangladeshi adults [12, 13]. A study based on BDHS 2011 found higher education, unemployment, and residents from the Khulna division possess a higher risk of HTN, DM, and HDC [14]. BDHS 2011 [15] was the first nationally representative survey that collected blood pressure and fasting glucose levels, which determines HTN and DM. Although it is quite outdated now, most of the existing studies on HTN and DM utilized this dataset. Some small-scale studies also reported prevalence and factors associated with HTN and DM among different communities in Bangladesh [16, 17]. However, a comprehensive trend analysis using the most recent nationally representative data (i.e., BDHS 2017–18) is lacking in Bangladesh. Besides, data on both HTN and DM combined (HDC) among Bangladeshi adults is nonexistent.

The government of Bangladesh developed a pragmatic action known as "Multisectoral Action Plan for Prevention and Control of Non—communicable Diseases 2018–2025" [18]. Early detection of NCD risk factors is crucial (action area—03) in this action plan. Further identification of risk factors is crucial to initiate immediate public health actions and allocate additional resources. Thus, it is essential to understand the current trend and progress to prevent and control the NCDs, particularly HTN and DM in Bangladesh. This study aimed to assess the trend in prevalence and associated factors of HTN, DM, and HDC among Bangladeshi adults from 2011–18. To our best knowledge, this is the first comprehensive trend analysis on HTN, DM, and HDC by using two waves of BDHS in Bangladesh. This study’s findings might help Bangladeshi policymakers to develop evidence-based interventions to control NCDs in Bangladesh.

## Methods

### Data source

This study was conducted on two nationally representative datasets called Bangladesh Demographic and Health Survey (BDHS): 2011 and 2017–18. BDHS is a national-level survey for Bangladeshi people that record a wide range of information on individuals’ demographic, socio-economic, and health characteristics since 1993. BDHS used two-stage stratified cluster sampling to collect data from selected households and surveyed through face-to-face interviews by trained staff. Details survey designs of BDHS have been published earlier in the final report of BDHS [10, 15].

We particularly used BDHS 2011 and BDHS 2017–18 since these datasets contain selected non-communicable diseases information (i.e., HTN & DM) [10]. BDHS collected information from 17,141 and 19,457 households with 83,731 and 89,819 members in 2011 and 2017–18, respectively. Totals of 3,733 males and 3,832 females aged ≥35 years attended blood pressure and glucose level measurements in BDHS 2011. Besides, 3,310 males and 3,518 females aged ≥18 years were available in the 2017–18 BDHS. We pooled the two BDHS datasets to generate a sufficiently large sample to precisely identify associated factors of HTN, DM, and HDC. This study included people aged 35 years and above as the BDHS 2011 does not contain NCD related information for people aged 18–34 years. After combining the two datasets and excluding the non-responders and missing, there were 11,686 adults as a study sample for the analysis.

### Outcome variable

This study considered three outcomes that were HTN, DM, and HTN-DM combined (HDC). The BDHS program provided a biomarker questionnaire to collect information about HTN and DM diagnosis and treatments. According to WHO’s recommended guidelines, this study measured systolic blood pressure (SBP), diastolic blood pressure (DBP), and plasma glucose levels. HTN was measured from systolic blood pressure (SBP) and diastolic blood pressure (DBP), whereas DM was measured from blood glucose levels. Trained health technicians recorded HTN data through Life Source UA-767 Plus BP monitor and DM data through HemoCue Glucose 201 Analyzer.

Three BP measurements were taken with 10-minute intervals. The average of the second and third measurements was utilized to calculate systolic blood pressure (SBP) and diastolic blood pressure (DBP). On the other hand, respondent’s blood was collected from the middle or ring finger to measure blood glucose after overnight fasting. The third blood drop measured blood glucose level while the first two drops were wiped away. Besides, plasma blood glucose level was obtained from blood glucose after adjustment [19]. And this whole procedure was much favored in developing countries to measure the blood glucose level [10, 15, 20].

The BDHS followed the American Heart Association (AHA) guidelines to measure cut-off points of HTN [12]. In this study, the presence of HTN was counted as if a respondent had systolic blood pressure (SBP) ≥ 140 mmHg (millimeters of mercury) or diastolic blood pressure (DBP) ≥ 90 mmHg and/or taking antihypertensive medication. Besides, BDHS applied the World Health Organization (WHO) cut-offs to measure plasma blood glucose levels [10, 15]. The fasting plasma glucose level was ≥7.0 mmol/L, which was reported as the presence of DM in this study. Here, prediabetes (PBG: 6.0–6.9 mmol/L with no medical care) and diabetes-free (PBG: < 6.0 mmol/L) categories were combined according to the BDHS classification scheme. We identified the respondents with HTN and DM simultaneously as HTN-DM combined (HDC) at the survey diagnosis time. All three outcomes followed binary measurements where value ’1’ represents ’having the disease,’ and ’0’ represents ’not having the disease.’

### Covariates

The following covariates were included in the study: age (35–39 years, 40–44 years, 45–49 years, 50–59 years, 60–69 years, ≥ 70 years), sex (male, female), education level (no formal education, primary, secondary, higher secondary and above), wealth status (poor, middle, rich), body mass index (normal, underweight, overweight), occupation type (physically inactive, physically active), diet plan (specified, random), drinking coffee (no, yes), place of residence (urban, rural), division (Barisal, Chittagong, Dhaka, Khulna, Rajshahi, Rangpur, Sylhet). Wealth status was extracted from the wealth index, which was computed by principal component analysis and ranged from poorest to richest [10, 15]. The wealth index is a composite measure of a household’s cumulative living standard. The wealth index is calculated using easy-to-collect data on a household’s ownership of selected assets, such as televisions and bicycles, materials used for housing construction, and types of water access and sanitation facilities. In our study, the stratification of wealth status considers poorest and poorer as poor and richer and richest as rich. Besides, the middle category of wealth status remains unchanged in the stratification. We organized body mass index into three categories: normal (BMI: 18.5–24.9), underweight (BMI <18.5), and overweight (BMI >24.9). Additionally, we generated the categories of occupation type for analytical purposes. We divided occupation into two categories based on the physical activity level: physically active (active workers) and physically inactive (inactive workers). For example, factory workers, boatmen, construction workers, rickshaw pullers, poultry raisers, cattle raisers, farmers, and agricultural workers were defined as active workers. Besides, retired persons, religious leaders, housewives, businessmen, and professionals were included as inactive workers. Moreover, we merged Mymensingh and Dhaka divisions as Dhaka for BDHS 2017–18 to match the dataset with BDHS 2011.

### Statistical analysis

This study covered univariate analysis through descriptive information, bivariate analysis through cross-tabulation with the chi-square test, and multivariate analysis through the modified Poisson regression with robust error variance. The number of persons having the disease per 100 adults aged ≥35 years was defined as the prevalence of selected NCDs (HTN, DM, and HDC). The prevalence of diseases for each baseline characteristic was obtained from the bivariate analysis. We showed the prevalence of selected NCDs at two survey time points separately. Additionally, rate of change analysis was adopted to evaluate the trend in HTN, DM, and HDC prevalence among adults aged 35 years and above by utilizing the two successive BDHS data (2011 & 2017–18). Hence, this study used the average annual rate of increase/ decrease to quantify the rate of change, and the formulas were acquired from UNICEF technical notes [21]. To assess the statistical significance of the trends, Cochran–Armitage test was applied [22, 23]. Then we presented the pooled prevalence of three diseases along with the chi-square test. The chi-square test shows the bivariate association between dependent and independent variables. Lastly, modified Poisson regression with robust error variance was applied to estimate the prevalence ratios of HTN, DM, and HDC with several socio-economic and demographic characteristics, respectively. Since the study contains two responses (HTN & DM) with prevalence greater than 10%, traditional binary logistic regression is unsuitable [24]. As a result, applying the logistic model could result in overestimated odds ratio (OR) with 95% confidence intervals (CIs). To avoid this problem, modelling with the Poisson technique could help to estimate the prevalence ratio. Although the Poisson model applies to rare events over time, the approach could overestimate the standard error (SE) of the prevalence ratio for the binomial dataset. Therefore, a substantial advantage of an alternative approach called the "modified Poisson regression with robust error variance" is used to estimate the error term precisely [25].

We utilized modified Poisson regression with robust error variance for three outcomes in three separate models. The PR, along with 95% confidence intervals, was exhibited in the table for interpretation purposes. We thoroughly utilized the sample weight to represent the data at the national level. Complete statistical analysis was executed through Stata (version 13) in this study. Variance Inflation Factor (VIF) was adopted to test the collinearity of the independent variables and the outcome variables (S2 Table).

#### Ethical approval

Data were extracted from Bangladesh Demographic and Health Survey (BDHS). NIPORT, Mitra and Associates, and ICF International conducted the survey by trainee staff [10, 15]. The interview was executed after verbal consent from each respondent based on an informed consent statement. ICF Institutional Review Board permitted the ethical approval for BDHS data according to Helsinki Declaration (1964) ethical standards [10, 15]. We accessed the data from https://dhsprogram.com/ after following the instructions and ensuring the data confidentiality deeds. The overall procedures like participants’ selection, sample size determination, sampling procedure, data preparation, ethical clearance, etc., are illustrated on their website and BDHS survey reports.

## Results

### Sociodemographic characteristics of the study participants

This study exhibits the pooled characteristics for the 11,686 study participants from BDHS: 2011 & 2017–18 (S3 Table). The highest percentage (25.24%) placed in the 50–59 years age group where the overall mean age of study participants was 52.79 years (SD±12.99). Among the participants, 42.28% were female, and 19.44% were overweight. The vast majority of the sample was physically inactive (60.16%) and belonged to poor households (48.85%). A large majority (76.24%) of the participants lived in the rural setting, and 31.16% resided in Dhaka.

### Trends of HTN, DM, and HDC

**Fig 1:**
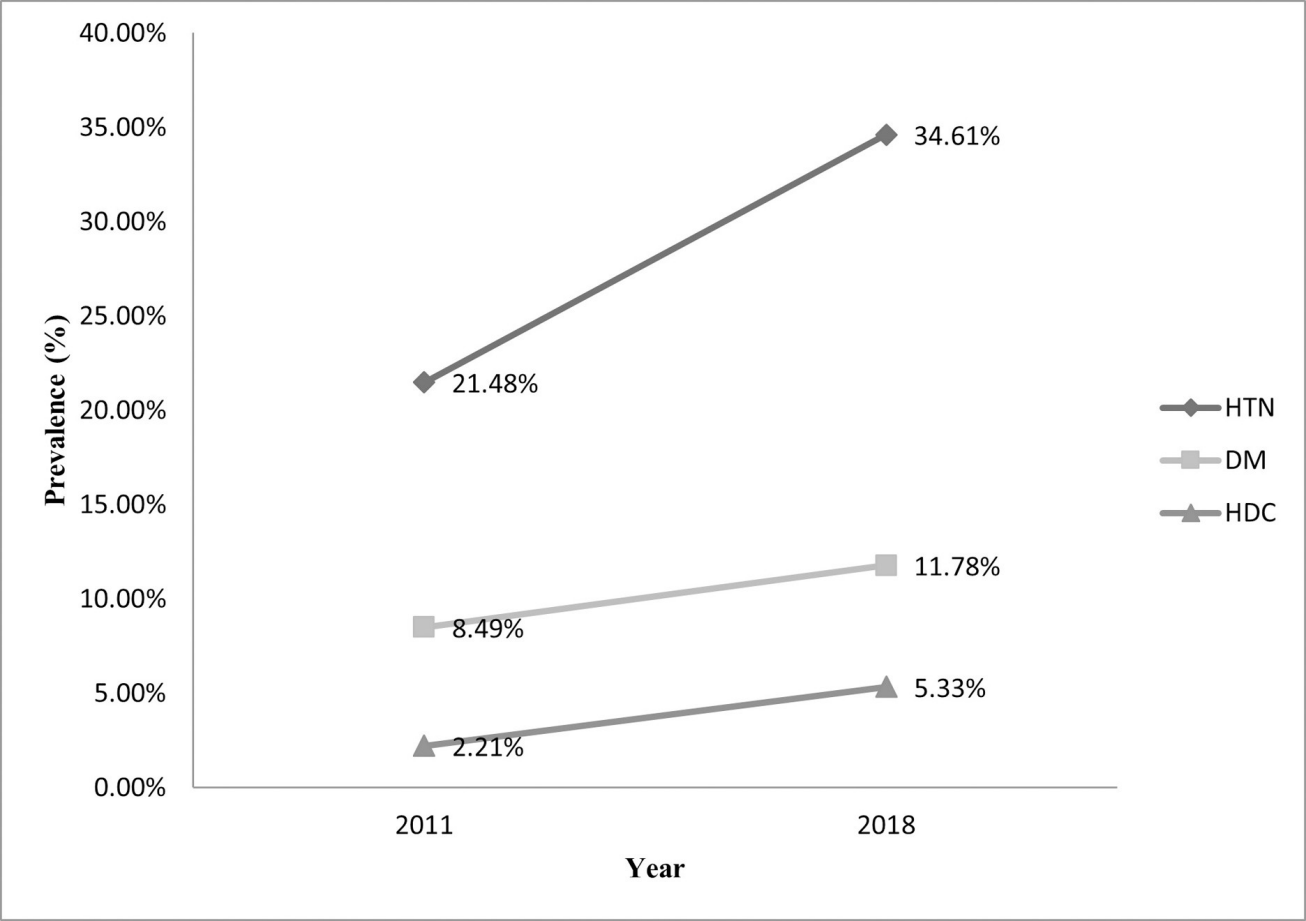
Prevalence of HTN, DM, and HDC among adults (≥ 35 years) in Bangladesh, BDHS (2011–2018).

Fig 1 displays the trends in the prevalence of HTN, DM, and HDC among adults in Bangladesh from 2011 to 2018. The prevalence of HTN among Bangladeshi adults has increased by 13 percentage points (from 21.48% in 2011 to 34.61% in 2018). Similarly, we found upward trends in DM and HDC prevalence, ranging from 8.49% & 2.21% (in 2011) to 11.78% & 5.33% (in 2018), respectively. Among the three outcomes, the prevalence of HTN was substantially higher over the periods. The increased trends of HTN, DM, and HDC were statistically significant (P<0.001) at a 5% level of significance (S4 Table).

Table 1 summarizes the prevalence of HTN, DM, and HDC among Bangladeshi adults by several characteristics. Compared to respective reference categories, the prevalence was higher among older adults, females, higher educated, rich, overweight, physically inactive, urban residents, specified diet takers, and coffee consumers. From 2011 to 2018, the upward trend of HTN prevalence has been shifted from the Khulna to the Barisal division. Besides, Chittagong and Dhaka had a higher prevalence of DM in 2011 and 2018, respectively. However, the prevalence of HDC among Chittagong residents was approximately double in 2018: 3.95% (2011) versus 6.59% (2018).

**Table 1:** The average annual rate of change of HTN, DM, and HDC prevalence among baseline characteristics in Bangladesh by year of survey.

Characteristics	Survey year 2011			Survey year 2017–18			Percent AARC (2011–2018)		
	HTN % (95% CI)	DM % (95% CI)	HDC % (95% CI)	HTN % (95% CI)	DM % (95% CI)	HDC % (95% CI)	HTN (P^3^)	DM (P^3^)	HDC (P^3^)
***Age***									
*35–39 years*	9.50 (7.41, 12.09)	5.34 (3.81, 7.42)	1.13 (0.55, 2.33)	24.36 (22.15, 26.70)	9.27(7.85, 10.92)	3.34 (2.51, 4.43)	14.45 (<0.001)	8.2 (<0.001)	16.74 (<0.001)
*40–44 years*	13.07 (10.52, 16.13)	6.70 (4.90, 9.90)	0.86 (0.36, 2.03)	26.68 (24.08, 29.45)	11.49 (9.69, 13.57)	4.39 (3.30, 5.81)	10.73 (0.002)	8.02 (0.263)	26.23 (0.198)
*45–49 years*	13.01 (10.52, 15.99)	8.60 (6.58, 11.15)	1.67 (0.90, 3.07)	32.46 (29.62, 35.43)	11.09 (9.28, 13.19)	5.59 (4.33, 7.20)	13.95 (<0.001)	3.7 (0.009)	18.84 (0.002)
*50–59 years*	22.42 (20.45, 24.52)	10.27 (8.89, 11.85)	2.83 (2.12, 3.76)	36.95 (34.40, 39.58)	14.00 (12.24, 15.96)	6.99 (5.74, 8.48)	7.4 (<0.001)	4.53 (<0.001)	13.79 (<0.001)
*60–69 years*	27.86 (25.16, 30.72)	7.97 (6.45, 9.82)	2.25 (1.50, 3.37)	42.86 (39.89, 45.89)	13.02 (11.11, 15.20)	5.57 (4.34, 7.13)	6.34 (<0.001)	7.26 (0.003)	13.83 (0.001)
≥ *70 years*	32.61 (29.48, 35.92)	9.07 (7.28, 11.24)	3.07 (2.09, 4.50)	51.77 (48.17, 55.36)	12.03 (9.88, 14.58)	6.67 (5.08, 8.70)	6.82 (<0.001)	4.11 (<0.001)	11.72 (<0.001)
***Sex***									
*Male*	16.16 (14.99, 17.41)	8.03 (7.18, 8.97)	1.82 (1.43, 2.32)	30.87 (29.29, 32.50)	11.63 (10.57, 12.80)	4.78 (4.09, 5.58)	9.69 (<0.001)	5.43 (<0.001)	14.79 (<0.001)
*Female*	33.36 (31.11, 35.71)	9.50 (8.16, 11.04)	3.09 (2.35, 4.60)	38.16 (36.53, 39.82)	11.92 (10.87, 13.07)	5.86 (5.11, 6.71)	1.94 (<0.001)	3.29 (0.118)	9.57 (0.003)
***Education level***									
*No formal education*	23.08 (21.46, 24.78)	6.90 (5.97, 7.97)	1.98 (1.50, 2.61)	35.26 (33.46, 37.11)	9.17 (8.13, 10.34)	4.12 (3.42, 4.94)	6.24 (0.003)	4.14 (<0.001)	11.04 (<0.001)
*Primary*	18.59 (16.60, 20.76)	8.71 (7.32, 10.34)	1.57 (1.03, 2.39)	32.02 (30.07, 34.06)	11.90 (10.58, 13.36)	4.64 (3.82, 5.63)	8.08 (0.010)	4.56 (<0.001)	16.74 (0.238)
*Secondary*	18.96 (16.52, 21.67)	8.90 (7.20, 10.95)	2.57 (1.72, 3.83)	36.65 (34.03, 39.36)	15.45 (13.56, 17.56)	7.49 (6.16, 9.08)	9.88 (0.829)	8.2 (<0.001)	16.51 (<0.001)
*Higher secondary and above*	26.26 (22.41, 30.52)	15.73 (12.66, 19.38)	4.72 (3.12, 7.10)	36.70 (32.76, 40.82)	15.49 (12.70, 18.76)	8.91 (6.80, 11.59)	4.9 (0.081)	**0.22**^**d**^ (<0.001)	9.5 (<0.001)
***Wealth status***									
*Poor*	18.13 (16.80, 19.54)	6.02 (5.23, 6.92)	1.38 (1.02, 1.86)	30.88 (29.16, 32.67)	6.49 (5.61, 7.49)	2.46 (1.93, 3.12)	7.91 (<0.001)	1.08 (<0.001)	8.61 (<0.001)
*Middle*	23.25 (20.82, 25.88)	8.11 (6.62, 9.90)	2.18 (1.46, 3.25)	34.91 (32.41, 37.50)	9.46 (8, 11.14)	4.32 (3.36, 5.55)	5.98 (<0.001)	2.22 (<0.001)	10.26 (<0.001)
*Rich*	29.41 (26.73, 32.24)	16.02 (13.93, 18.36)	4.66 (3.55, 6.11)	38.40 (36.51, 40.31)	18.63 (17.16, 20.20)	8.92 (7.87, 10.10)	3.88 (<0.001)	2.18 (<0.001)	9.72 (<0.001)
***Body mass index (BMI)***									
*Normal (18*.*5–24*.*9)*	21.91 (20.47, 23.43)	8.50 (7.56, 9.56)	2.32 (1.84, 2.93)	32.45 (30.95, 33.99)	10.55 (9.59, 11.59)	4.49 (3.86, 5.21)	5.77 (<0.001)	3.14 (<0.001)	9.89 (<0.001)
*Underweight(<18*.*5)*	16.56 (14.83, 18.45)	5.23 (4.25, 6.43)	0.71 (0.40, 1.25)	23.87 (21.53, 26.38)	6.53 (5.26, 8.08)	1.54 (0.98, 2.41)	5.36 (<0.001)	3.22 (<0.001)	11.69 (<0.001)
*Overweight (>24*.*9)*	33.46 (29.67, 37.48)	17.83 (14.88, 21.23)	6.00 (4.31, 8.29)	46.60 (44.25, 48.97)	18.02 (16.28, 19.91)	9.73 (8.42, 11.23)	4.84 (<0.001)	0.15 (<0.001)	7.15 (<0.001)
***Occupation type***									
*Physically active*	11.60 (10.20, 13.15)	5.17 (4.24, 6.29)	0.91 (0.56, 1.46)	28.26 (26.64, 29.94)	7.49 (6.58, 8.52)	2.70 (2.16, 3.36)	13.56 (<0.001)	5.44 (<0.001)	16.81 (<0.001)
*Physically inactive*	26.80 (25.33, 28.33)	10.27 (9.29, 11.34)	2.92 (2.40, 3.54)	39.54 (37.97, 41.13)	15.11 (13.99, 16.30)	7.38 (6.58, 8.27)	5.72 (<0.001)	5.68 (<0.001)	14.17 (<0.001)
***Having HTN***									
*No*	*-*	7.99 (7.19, 8.86)	*-*	*-*	9.86 (9, 10.80)	*-*		3.05 (<0.001)	
*Yes*	*-*	10.31 (8.66, 12.24)	*-*	*-*	15.41 (13.98, 16.96)	*-*		5.91 (<0.001)	
***Having DM***									
*No*	21.05 (19.92, 22.24)	*-*	*-*	33.19 (31.98, 34.41)	*-*	*-*	6.72 (<0.001)		
*Yes*	26.10 (22.21, 30.41)	*-*	*-*	45.26 (41.77, 48.80)	*-*	*-*	8.18 (<0.001)		
***Diet plan***									
*Random*	20.33 (18.29, 22.55)	7.30 (6.04, 8.80)	1.91 (1.31, 2.78)	33.96 (32.02, 35.95)	11.20 (9.96, 12.58)	4.91 (4.09, 5.89)	7.61 (0.542)	6.3 (0.236)	14.4 (0.966)
*Specified*	21.90 (20.61, 23.24)	8.91 (8.05, 9.86)	2.32 (1.89, 2.85)	34.95 (33.54, 36.39)	12.08 (11.14, 13.09)	5.55 (4.90, 6.28)	6.91 (<0.001)	4.45 (<0.001)	13.27 (<0.001)
***Drinking coffee***									
*No*	21.48 (20.36, 22.65)	8.22 (7.48, 9.02)	2.21 (1.83, 2.66)	34.48 (33.29, 35.69)	11.56 (10.78, 12.39)	5.12 (4.60, 5.71)	6.99 (<0.001)	4.99 (<0.001)	12.75 (<0.001)
*Yes*	21.49 (16.92, 26.90)	13.60 (9.95, 18.32)	2.35 (1.09, 5.01)	36.31 (32.13, 40.70)	14.56 (11.69, 17.99)	7.96 (5.86, 10.73)	7.78 (0.061)	0.97 (0.006)	19.04 (0.014)
***Place of residence***									
*Urban*	26.18 (23.74, 28.77)	12.05 (10.31, 14.04)	3.62 (2.69, 4.85)	36.89 (34.57, 39.28)	16.34 (14.61, 18.23)	7.66 (6.46, 9.07)	5.02 (<0.001)	4.45 (<0.001)	11.3 (<0.001)
*Rural*	20.11 (18.89, 21.38)	7.44 (6.67, 8.30)	1.80 (1.43, 2.26)	33.87 (32.56, 35.20)	10.29 (9.47, 11.17)	4.57 (4.02, 5.19)	7.73 (<0.001)	4.74 (<0.001)	14.24 (<0.001)
***Division***									
*Barisal*	19.52 (15.43, 24.38)	9.97 (7.07, 13.89)	2.30 (1.11, 4.69)	39.32 (34.58, 44.28)	9.79 (7.21, 13.16)	3.59 (2.15, 5.96)	10.52 (<0.001)	**0.26**^**d**^ (<0.001)	6.57 (0.016)
*Chittagong*	17.00 (14.65, 19.64)	12.15 (10.15, 14.49)	3.95 (2.85, 5.46)	35.37 (32.55, 38.31)	14.44 (12.45, 16.69)	6.59 (5.24, 8.25)	11.04 (0.006)	2.5 (0.062)	7.58 (0.027)
*Dhaka*	21.79 (19.88, 23.83)	7.78 (6.59, 9.16)	1.91 (1.36, 2.69)	30.55 (28.55, 32.62)	15.40 (13.87, 17.06)	6.53 (5.55, 7.71)	4.95 (0.108)	10.24 (0.648)	19.2 (0.210)
*Khulna*	28.45 (25.20, 31.94)	5.78 (4.27, 7.79)	1.76 (1.01, 3.05)	36.71 (33.62, 39.91)	9.43 (7.69, 11.52)	5.33 (4.04, 7)	3.71 (0.403)	7.24 (0.025)	17.15 (0.665)
*Rajshahi*	19.14 (16.39, 22.23)	8.86 (6.97, 11.2)	2.02 (1.21, 3.36)	35.90 (32.90, 39.01)	9.95 (8.20, 12.02)	4.23 (3.12, 5.71)	9.41 (0.026)	1.67 (0.180)	11.14 (0.663)
*Rangpur*	24.88 (21.69, 28.36)	7.02 (5.29, 9.26)	1.55 (0.85, 2.84)	37.90 (34.74, 41.12)	6.56 (5.10, 8.41)	3.09 (2.13, 4.46)	6.19 (<0.001)	**0.97**^**d**^ (<0.001)	10.36 (0.331)
*Sylhet*	16.89 (13.03, 21.62)	8.75 (6.02, 12.56)	1.65 (0.70, 3.87)	33.12 (28.71, 37.86)	9.93 (7.38, 13.24)	5.31 (3.52, 7.96)	10.1 (<0.001)	1.83 (0.480)	18.18 (0.655)

CI = Confidence interval

HTN = Hypertension

DM = Diabetes mellitus

HDC = HTN-DM combined

AARC = Average annual rate of change

AARC^d^ = Average annual rate of change (decrease)

P^3^ **=** *P*-value for the Cochran–Armitage test of percent AARC from the year 2011.

Table 1 also reports the average annual rate of change of HTN, DM, and HDC prevalence to measure the exact rate of increase/ decrease of the prevalence among selected background characteristics in Bangladesh. The average annual rates had increased for all considered socio-economic and demographic characteristics of HTN and HDC prevalence except DM prevalence. Among all the covariates, statistically significant annual decreases of DM prevalence had been identified among adults from higher education (0.22%), Barisal (0.26%), and Rangpur (0.97%) residents. These reported decrease rates in the prevalence of DM were relatively very minimum number. Except for these three categories, the annual rates in the prevalence of HTN, DM, and HDC had substantially increased for all covariates between 2011 and 2018.The pooled prevalence of the study population is presented in Table 2 over the periods 2011–2018. The prevalence of HTN and HDC increased with age among adults. Besides, DM prevalence was highest in the age group 50–59 years. The younger-older differences were excessive among adults having HTN. The prevalence of three diseases was higher among females and higher educated than males and uneducated people over the periods 2011–2018. HTN, DM, and HDC prevalence varied notably by wealth status among Bangladeshi adults. Overweight adults had the highest prevalence of HTN, DM, and HDC. Differences in prevalence of HTN, DM, and HDC were higher among physically inactive adults. The reported prevalence of HTN, DM, and HDC was comparatively higher among the adults who followed a specified diet and drank coffee. Table 2 also revealed the higher prevalence of HTN, DM, and HDC among urban than rural residents. HTN and DM prevalence varied substantially among the divisions. In contrast, the prevalence of HDC was slow-going among divisions in Bangladesh. All the covariates except diet plan and drinking coffee were significantly associated with HTN. On the other, all the independent variables but diet plan had a significant bivariate association with DM and HDC among Bangladeshi adults.

**Table 2:** The pooled weighted prevalence of HTN, DM, and HDC by selected covariates with Chi-square test in Bangladesh (2011–2018).

Characteristics	Total n (%)	HTN % (95% CI)	P-value	DM % (95% CI)	P-value	HDC % (95% CI)	P-value
***Age***							
*35–39 years*	1,975 (16.90)	19.79 (18.09, 21.60)	<0.001	8.06 (6.94, 9.35)	0.001	2.66 (2.04, 3.47)	0.001
*40–44 years*	1,595 (13.65)	21.94 (19.98, 24.04)		9.82 (8.46, 11.38)		3.16 (2.41, 4.14)	
*45–49 years*	1,577 (13.49)	25.28 (23.20, 27.48)		10.17 (8.77, 11.76)		4.14 (3.27, 5.23)	
*50–59 years*	2,949 (25.24)	29.00 (27.39, 30.66)		11.96 (10.83, 13.18)		4.71 (4.01, 5.54)	
*60–69 years*	2,039 (17.45)	35.54 (33.49, 37.64)		10.56 (9.30, 11.97)		3.95 (3.19, 4.89)	
≥ *70 years*	1,551 (13.26)	41.75 (39.32, 44.22)		10.48 (9.05, 12.11)		4.79 (3.83, 5.97)	
***Sex***							
*Male*	6,746 (57.72)	23.09 (22.10, 24.11)	<0.001	9.73 (9.04, 10.46)	0.003	3.21 (2.82, 3.66)	<0.001
*Female*	4,940 (42.28)	36.61 (35.28, 37.97)		11.14 (10.29, 12.05)		4.97 (4.39, 5.61)	
***Education level***							
*No formal education*	5,119 (43.80)	29.34 (28.11, 30.61)	0.019	8.07 (7.36, 8.85)	<0.001	3.08 (2.64, 3.59)	<0.001
*Primary*	3,429 (29.34)	26.77 (25.32, 28.28)		10.65 (9.66, 11.73)		3.44 (2.88, 4.10)	
*Secondary*	2,141 (18.32)	29.31 (27.42, 31.28)		12.73 (11.39, 14.21)		5.45 (4.56, 6.49)	
*Higher secondary and above*	997 (8.53)	31.99 (29.17, 34.95)		15.60 (13.48, 17.98)		7.02 (5.59, 8.78)	
***Wealth status***							
*Poor*	5,709 (48.85)	24.08 (22.98, 25.20)	<0.001	6.24 (5.64, 6.90)	<0.001	1.88 (1.56, 2.27)	<0.001
*Middle*	2,412 (20.64)	29.76 (27.97, 31.61)		8.86 (7.79, 10.06)		3.38 (2.73, 4.18)	
*Rich*	3,565 (30.50)	35.75 (34.19, 37.34)		17.86 (16.64, 19.15)		7.66 (6.84, 8.58)	
***Body mass index (BMI)***							
*Normal (18*.*5–24*.*9)*	6,614 (56.60)	27.68 (26.61, 28.77)	<0.001	9.62 (8.93, 10.36)	<0.001	3.51 (3.09, 3.98)	<0.001
*Underweight (<18*.*5)*	2,801 (23.97)	19.65 (18.22, 21.17)		5.78 (4.98, 6.71)		1.06 (0.74, 1.51)	
*Overweight (>24*.*9)*	2,271 (19.44)	43.38 (41.36, 45.43)		17.98 (16.45, 19.61)		8.82 (7.72, 10.06)	
***Occupation type***							
*Physically active*	4,655 (39.84)	21.79 (20.63, 23.00)	<0.001	6.59 (5.91, 7.34)	<0.001	2.00 (1.64, 2.45)	<0.001
*Physically inactive*	7,031 (60.16)	33.46 (32.36, 34.57)		12.80 (12.04, 13.60)		5.25 (4.75, 5.79)	
***Having HTN***							
*No*	8,319 (71.19)	*-*	*-*	8.95 (8.35, 9.58)	<0.001	*-*	*-*
*Yes*	3,367 (28.81)	*-*		13.73 (12.61, 14.93)		*-*	
***Having DM***							
*No*	10,479 (89.67)	27.72 (26.87, 28.58)	<0.001	*-*	*-*	*-*	*-*
*Yes*	1,207 (10.33)	38.30 (35.60, 41.08)		*-*		*-*	
***Diet plan***							
*Random*	3,602 (30.82)	28.76 (27.31, 30.26)	0.905	9.72 (8.79, 10.73)	0.231	3.77 (3.20, 4.44)	1.000
*Specified*	8,084 (69.18)	28.83 (27.85, 29.83)		10.60 (9.94, 11.29)		4.04 (3.63, 4.45)	
***Drinking coffee***							
*No*	10,947 (93.68)	28.65 (27.81, 29.51)	0.277	10.06 (9.52, 10.64)	<0.001	3.82 (3.48, 4.19)	0.008
*Yes*	739 (6.32)	31.13 (27.90, 34.56)		14.22 (11.89, 16.93)		6.00 (4.51, 7.95)	
***Place of residence***							
*Urban*	2,777 (23.76)	32.38 (30.66, 34.14)	<0.001	14.54 (13.27, 15.90)	<0.001	5.96 (5.14, 6.90)	<0.001
*Rural*	8,909 (76.24)	27.70 (26.78, 28.64)		9.01 (8.44, 9.63)		3.33 (2.98, 3.72)	
***Division***							
*Barisal*	686 (5.87)	30.66 (27.32, 34.21)	<0.001	9.87 (7.85, 2.33)	<0.001	3.03 (1.98, 4.60)	0.012
*Chittagong*	1,924 (16.46)	27.06 (25.13, 29.09)		13.41 (1.96, 15)		5.39 (4.47, 6.50)	
*Dhaka*	3,642 (31.16)	26.51 (25.10, 27.97)		11.89 (10.88, 12.98)		4.41 (3.79, 5.12)	
*Khulna*	1,582 (13.54)	33.13 (30.86, 35.49)		7.85 (6.63, 9.28)		3.79 (2.95, 4.85)	
*Rajshahi*	1,644 (14.06)	28.78 (26.64, 31.02)		9.49 (8.16, 11)		3.29 (2.53, 4.27)	
*Rangpur*	1,514 (12.95)	32.37 (30.06, 34.77)		6.76 (5.60, 8.13)		2.43 (1.77, 3.34)	
*Sylhet*	694 (5.95)	26.31 (23.17, 29.71)		9.44 (7.48, 11.84)		3.78 (2.60, 5.47)	

CI = Confidence interval.

HTN = Hypertension.

DM = Diabetes mellitus.

HDC = HTN-DM combined.

Table 3 presents the adjusted PRs for HTN, DM, and HDC for the predictors. The modified Poisson regression with robust error variance suggested that physically inactive workers, overweight adults, specified diet takers, adults in wealthy households, and the older age group had, in general, significantly higher diseases prevalence. Compared to younger, older adults had a significantly higher prevalence of developing the diseases. The risk of HTN and HDC in adults aged ≥ 70 years was 2.70 (PR: 2.70; 95% CI: 2.42–3.00) and 2.97 (PR: 2.97; 95% CI: 2.08–4.24) times higher, respectively, compared to adults aged 35–39 years. Besides, adults aged 50–59 years had (OR: 1.82; 95% CI: 1.48–2.23) a higher prevalence of getting DM (PR: 1.67; 95% CI: 1.40–1.99) than 35–39 years adults.

**Table 3:** Estimated prevalence ratios (PR) with 95% confidence intervals for HTN, DM, and HDC from modified Poisson regression with robust error variance among adults (≥ 35 years) in Bangladesh (2011–2018).

*Characteristics*	HTN			DM			HDC		
	PR	(95% CI)	P-value	PR	(95% CI)	P-value	PR	(95% CI)	P-value
***Age***									
*35–39 years (RC)*									
*40–44 years*	1.19^b^	(1.06, 1.35)	0.004	1.25^b^	(1.02, 1.53)	0.034	1.32	(0.91, 1.92)	0.143
*45–49 years*	1.39^a^	(1.24, 1.56)	<0.001	1.30^b^	(1.06, 1.59)	0.013	1.80^a^	(1.27, 2.54)	0.001
*50–59 years*	1.73^a^	(1.56, 1.91)	<0.001	1.67^a^	(1.40, 1.99)	<0.001	2.54^a^	(1.86, 3.45)	<0.001
*60–69 years*	2.15^a^	(1.94, 2.39)	<0.001	1.55^a^	(1.27, 1.89)	<0.001	2.20^a^	(1.56, 3.10)	<0.001
≥ *70 years*	2.70^a^	(2.42, 3.00)	<0.001	1.59^a^	(1.28, 1.97)	<0.001	2.97^a^	(2.08, 4.24)	<0.001
***Sex***									
*Male (RC)*									
*Female*	1.31^a^	(1.23, 1.39)	<0.001	1.02	(0.91, 1.15)	0.730	1.23^b^	(1.01, 1.50)	0.036
***Education level***									
*No formal education (RC)*									
*Primary*	0.98	((0.92, 1.05)	0.642	1.18^b^	(1.03, 1.36)	0.020	0.98	(0.78, 1.24)	0.886
*Secondary*	1.04	(0.96, 1.13)	0.302	1.19^b^	(1.01, 1.39)	0.038	1.19	(0.92, 1.54)	0.190
*Higher secondary and above*	1.05	(0.94, 1.18)	0.356	1.16	(0.96, 1.41)	0.127	1.31^c^	(0.96, 1.78)	0.092
***Wealth status***									
*Poor (RC)*									
*Middle*	1.08^c^	(1.00, 1.16)	0.047	1.18^c^	(0.99, 1.40)	0.059	1.31^c^	(0.96, 1.77)	0.085
*Rich*	1.10^b^	(1.02, 1.19)	0.017	1.96^a^	(1.68, 2.29)	<0.001	2.07^a^	(1.57, 2.71)	<0.001
***Body mass index (BMI)***									
*Normal (18*.*5–24*.*9) (RC)*									
*Underweight (<18*.*5)*	0.70^a^	(0.65, 0.76)	<0.001	0.69^a^	(0.58, 0.82)	<0.001	0.32^a^	(0.22, 0.48)	<0.001
*Overweight (>24*.*9)*	1.41^a^	(1.33, 1.51)	<0.001	1.47^a^	(1.30, 1.66)	<0.001	1.87^a^	(1.54, 2.28)	<0.001
***Occupation type***									
*Physically active (RC)*									
*Physically inactive*	1.23^a^	(1.16, 1.33)	<0.001	1.35^a^	(1.18, 1.56)	<0.001	1.52^a^	(1.18, 1.92)	0.001
***Having HTN***									
*No (RC)*									
*Yes*	NA	NA	NA	1.19^a^	(1.07, 1.33)	0.002	NA	NA	NA
***Having DM***									
*No (RC)*									
*Yes*	1.12^a^	(1.04, 1.21)	0.003	NA	NA	NA	NA	NA	NA
***Diet Plan***									
*Random (RC)*									
*Specified*	1.10^a^	(1.04, 1.18)	0.002	1.18^b^	(1.04, 1.33)	0.011	1.20^c^	(0.98, 1.47)	0.081
***Drinking coffee***									
*No (RC)*									
*Yes*	1.06	(0.95, 1.17)	0.319	1.27^b^	((1.05, 1.54)	0.012	1.25	(0.92, 1.69)	0.153
***Place of residence***									
*Urban (RC)*									
*Rural*	0.99	(0.93, 1.05)	0.697	0.99	(0.88, 1.11)	0.805	0.98	(0.81, 1.18)	0.801
***Division***									
*Barisal (RC)*									
*Chittagong*	0.84^a^	(0.75, 0.93)	0.001	1.05	(0.87, 1.28)	0.601	1.19	(0.84, 1.69)	0.315
*Dhaka*	0.87^a^	(0.79, 0.96)	0.007	1.00	(0.83, 1.21)	0.996	1.08	(0.77, 1.50)	0.652
*Khulna*	1.07	(0.97, 1.18)	0.183	0.75^a^	(0.61, 0.92)	0.006	0.99	(0.70, 1.41)	0.976
*Rajshahi*	1.03	(0.93, 1.15)	0.537	0.91	(0.74, 1.13)	0.413	1.00	(0.69, 1.45)	0.994
*Rangpur*	1.21^a^	(1.09, 1.34)	<0.001	0.75^b^	(0.60, 0.95)	0.015	0.93	(0.63, 1.38)	0.717
*Sylhet*	0.87^b^	(0.78, 0.98)	0.024	0.93	(0.75, 1.15)	0.483	1.09	(0.74, 1.58)	0.669
***Survey year***									
*2011 (RC)*									
*2017–18*	1.55^a^	(1.46, 1.65)	<0.001	1.05	(0.93, 1.17)	0.453	1.86^a^	(1.50, 2.30)	<0.001

RC = Reference category.

PR = Prevalence ratio.

CI = Confidence interval.

HTN = Hypertension.

DM = Diabetes mellitus.

HDC = HTN-DM combined.

*P*-value:

a*<* 0.01

b*<* 0.05, and

c < 0.10.

Regarding wealth status, the risks of three diseases were higher among rich adults than poor adults. Here, the prevalence of getting DM and HDC were almost similar among rich adults. Overweight adults had a significantly higher prevalence of having HTN (PR: 1.41; 95% CI: 1.33–1.51), DM (PR: 1.47; 95% CI: 1.30–1.66), and HDC (PR: 1.87; 95% CI: 1.54–2.28), respectively, compared to normal weighted people in Bangladesh. When comparing physically active and inactive occupation, the prevalence for inactive adults were 23% (PR: 1.23; 95% CI: 1.16–1.33), 35% (PR: 1.35; 95% CI: 1.18–1.56), and 52% (PR: 1.52; 95% CI: 1.18–1.92) higher for developing HTN, DM, and HDC, respectively.

The education level of adults was significantly associated with the risk of DM and HDC. The HTN (PR: 1.31; 95% CI: 1.23–1.39) and HDC (PR: 1.23; 95% CI: 1.01–1.50) prevalence were higher among females compared to males. Adults with DM (PR: 1.12; 95% CI: 1.04–1.21) were more likely to diagnose HTN than adults without DM. A similar result was observed between having HTN and DM. Besides, drinking coffee was only associated with the risk of DM. Coffee drinker adults had a higher prevalence of developing DM (PR: 1.27; 95% CI: 1.05–1.54) compared to non-drinker. The divisions had a significant impact on the risk of HTN and DM. The prevalence of developing HTN and DM showed substantial geographical variations among adults. Additionally, the probability of developing three diseases was increased among adults over the period. The prevalence of getting HTN and HDC was increased by 55% and 86% (in 2017–18), respectively, compared to the survey year 2011. On the other hand, the likelihood of developing DM increased by 5% (in 2017–18) compared to the survey year 2011.

## Discussion

Our study shows the prevalence, trends, and associated factors of HTN, DM, and HDC in Bangladesh using the most recent and previous nationally representative surveys. The prevalence of HTN among Bangladeshi adults increased by 13 percentage points, from 21.48% to 34.61% between 2011 to 2018. We found rising trends in DM and HDC prevalence, shifting from 8.49% and 2.21% (in 2011) to 11.78% and 5.33% (in 2018), respectively. Besides, all the covariates have increased the average annual rate (AAR) in the prevalence of HTN and HDC from 2011 to 2018. The prevalence of HDC was higher and nearly doubled in Chittagong consistent with a previous study in Bangladesh using the BDHS2011 data [14]. This could be explained by the rapid urbanization and industrialization in Chittagong with uprising labor force in the manufacturing sector rather than the primary sector [26]. The decreased physical activity levels among people in Chittagong lead them to live a sedentary life. Overall, the covariates, i.e., occupation type, BMI, wealth status, diet plan, and age was, in general, significant factors that increased the prevalence of HTN, DM, and HDC and is identical to the findings from developing countries like Bangladesh [12, 14, 27–29].

This study demonstrates that older adults have a greater prevalence and likelihood of having HTN, DM, and HDC than younger adults. Evidence from several studies shows the existence of a significant positive association between age and HTN and DM: as age increases, the incidence of HTN and DM increases [30–34]. A flawed immunity system, low nutritional status, less physical activity may be the main reasons for this suffering of older people in Bangladesh [35].

Regarding wealth status, the risks of HTN, DM, and HDC were higher among affluent adults than poor adults. We found that wealth status was positively associated with HTN, DM, and HDC from previous studies. Respondents from the highest wealth quintile have a higher chance of developing HTN, DM, and HDC than those of the lowest wealth quintile [14, 32, 33]. People from the higher wealth quintile are more likely to engage in sedentary lifestyles and less physical labor, leading them to be overweight/obese and have more chance to develop HTN, DM, and HDC [36]. An inverse relationship exists between HTN and education in rural East Asia and a positive association in rural South Asia indicated by a recent meta-analysis [37]. Our study shows education had an association with the risk of DM and HDC as it increases the living standard. Higher educated adults are more at risk of developing DM, and HDC, supported by previous studies [14, 27, 32, 38]. In general, people with higher education have better chances for higher income and become wealthier, making their work environment comfortable. With increased revenue, wealthy people can spend their money for more comfort by reducing their physical activities and exercise, increasing the chance to develop DM, and HDC. Moreover, workload and mental pressure are also high for wealthy people, which provoke them to lead a stressful life [39].

Our study demonstrates that overweight adults had a higher probability of developing HTN, DM, and HDC than adults who had normal weight in Bangladesh. Previous studies found that overweight/obesity (higher BMI) is associated with a higher prevalence of HTN, DM, and HDC [33, 40–42]. An inverse relationship between coffee consumption and the risk of DM was reported in a Japanese study [43]. Besides, an American study did not find any substantial association between coffee consumption and the risk of DM [44]. However, such findings were not observed in our study. Our results suggested that drinking coffee was related to the risk of DM in Bangladeshi adults. Adults who drink coffee had a higher likelihood of developing DM than those who did not drink coffee. Previous studies mentioned evidence that the higher rate of coffee intake was related to high BMI, less exercise, and an unhealthy diet [45, 46]. Thus such confounding by dietary and lifestyle factors might be the probable reason for the discrepancy between previous findings and our findings. [45]. However, further investigation is needed in this regard among Bangladeshi adults. Consumption of energy-dense foods and a sedentary lifestyle may lead individuals to develop HTN conditions [47, 48]. The probability of developing HTN, DM, and HDC was lower among physically active adults than physically inactive adults [35, 49]. Several studies noted moderate or high physical activities were inversely related to DM and HTN prevalence [50]. Physical activity like walking, swimming, and exercise provide health benefits among high-risk adults, including HTN, DM, HDC, and high BMI [51, 52]. Adopting a healthier lifestyle, including healthier food habits, avoiding alcohol, smoking, and maintaining regular exercise, could help Bangladeshi people keep their BMI within a standard range to control HTN and/or DM risk.

The current study shows that females had substantially higher prevalence of developing HTN and HDC than male adults. The likelihood of female adults developing HTN increases as they grow older and become more significant than men [32, 34, 38]. Due to different biological and environmental factors, women may have a higher chance of having HTN and HDC. Moreover, mental stress and anxiety are related to HTN, and women may be more exposed to stress and anxiety due to their biological factors like menopause [53, 54]. Our results contrast with an Indian study, where males had a higher probability of developing HTN than females [55]. We did not find any association between sex and the risk of developing DM. A previous study in Bangladesh also reported that sex was not associated with DM [14].

Contrary to the finding, a recent study found sex had a significant influence on developing DM [33]. However, this dissimilarity has been observed in the results due to changes in data structure and methodology. Our research found a significant association with the risk of HTN and DM with regional differences consistent with some previous studies. Studies showed that regional variations are significantly associated with HTN and DM prevalence [33, 34, 56–58]. Additionally, the risk of developing HTN, DM, and the coexistence of DM and HTN were higher among adults over 2011–2018 in Bangladesh. Some existing studies revealed that the prevalence of HTN and DM is increasing worldwide [47, 59].

### Strengths and limitations

This study has numerous drawbacks. We have studied the association of only those variables that were available in the BDHS data set. This study could not include several factors like a family history of DM and HTN, dietary practice, lifestyle, smoking behavior, alcohol consumption, and medication history due to data unavailability. Along with the biopsychosocial conditions, a temporal effect on variability can be observed due to the large time gap between the sample data collections. Despite these limitations, this study’s main strength is that it involves two nationally representative samples for Bangladesh’s adult population.

This study shows the year-specific prevalence of HTN, DM, and HDC using two updated BDHS waves (2011 & 2017–18). Rate of change analysis is a new approach to measure the change (increase/decrease) of HTN, DM, and HDC prevalence by the baseline characteristics from 2011 to 2018. A pooled dataset identifies several contemporary socio-demographic and socioeconomic characteristics associated with HTN, DM, and HDC.

## Conclusions

The study shows that from 2011 to 2018, the prevalence of HTN, DM, and HDC has increased by 13, 3.29, and 3.12 percentage points, respectively. Inactive workers, overweight adults, adults from wealthy families, and the elderly group had a comparatively higher prevalence of developing HTN, DM, and HDC. Additionally, drinking coffee is a significant risk factor for DM in Bangladesh. This study’s findings have significant implications because HTN, DM, and HDC are highly prevalent in Bangladesh. Health strategies endorsed by the government and non-government organizations might help adults with HTN or DM or HDC target groups control the severity of the disease. Therefore, health programs based on risk factor prevention could help to reduce the disease prevalence. Initiatives to spread awareness among people at an early age are needed, including special attention for older people and those at risk for NCDs. Other effective interventions include maintaining body weight, encouraging physical activity, and leading a healthy lifestyle.

## Supporting information

S1 Table STROBE checklist. (DOC)

S2 Table Test results of Variance Inflation Factor (VIF). (DOCX)

S3 Table Baseline characteristics of the study participants aged 35 and over from Bangladesh demographic and health survey (n = 11,686). (DOCX)

S4 Table Test results of Cochran-Armitage test. (DOCX)

## Abbreviation

NCDs: Non-communicable diseases
HTN: Hypertension
DM: Diabetes mellitus
HDC: HTN-DM combined
BMI: Body mass index
