# Supplementary material for: Prevalence, trends and associated factors of hypertension and diabetes mellitus in Bangladesh: Evidence from BHDS 2011 and 2017–18
Source: PLoS One. 2022 May 3;17(5):e0267243. doi: 10.1371/journal.pone.0267243 (PMC9064112; doi:10.1371/journal.pone.0267243)
Supplement: S1 Table — (DOC) [file pone.0267243.s001.doc]

**S1 Table. STROBE checklist**

| **Section/Topic** | Item No | Recommendation | Reported Page Number |
| --- | --- | --- | --- |
| **Title and abstract** | 1 | (*a*) Indicate the study’s design with a commonly used term in the title or the abstract | Please refer to the page 1, lines 35 to 44. |
|  |  | (*b*) Provide in the abstract an informative and balanced summary of what was done and what was found | Please refer to the page 1 & 2, lines 38 to 57. |
| Introduction | | |  |
| Background/rationale | 2 | Explain the scientific background and rationale for the investigation being reported | Please refer to the page 3 & 4, lines 69 to 104. |
| Objectives | 3 | State specific objectives, including any prespecified hypotheses | Please refer to the page 4, lines 110 to 112. |
| Methods | | |  |
| Study design | 4 | Present key elements of study design early in the paper | Please refer to the page 4 & 5, lines 110 to 115. |
| Setting | 5 | Describe the setting, locations, and relevant dates, including periods of recruitment, exposure, follow-up, and data collection | Please refer to the page 5, lines 118 to 126. |
| Participants | 6 | (*a*) Give the eligibility criteria, and the sources and methods of selection of participants | Please refer to the page 5-6 & 9-10, lines 118 to 134 and lines 216 to 224. |
| Variables | 7 | Clearly define all outcomes, exposures, predictors, potential confounders, and effect modifiers. Give diagnostic criteria, if applicable | Please refer to the page 6 to 8, lines 136 to184. |
| Data sources/ measurement | 8* | For each variable of interest, give sources of data and details of methods of assessment (measurement). Describe comparability of assessment methods if there is more than one group | Please refer to the page 6 to 8, lines 136 to184. |
| Bias | 9 | Describe any efforts to address potential sources of bias | Please refer to the page 25, lines 411 to 416. |
| Study size | 10 | Explain how the study size was arrived at | Please refer to the page 5 to 6, lines 126 to 134. |
| Quantitative variables | 11 | Explain how quantitative variables were handled in the analyses. If applicable, describe which groupings were chosen and why | Please refer to the page 6, 7 & 8, lines 136 to 161, lines 163 to 164, and lines 175 to 177. |
| Statistical methods | 12 | (*a*) Describe all statistical methods, including those used to control for confounding | Please refer to the page 8 - 10, lines 186 to 214. |
|  |  | (*b*) Describe any methods used to examine subgroups and interactions | N/A |
|  |  | (*c*) Explain how missing data were addressed | Please refer to the page 6, lines 133 to 134. |
|  |  | (*d*) If applicable, describe analytical methods taking account of sampling strategy | N/A |
|  |  | (*e*) Describe any sensitivity analyses | N/A |
| Results | | |  |
| Participants | 13* | (a) Report numbers of individuals at each stage of study—eg numbers potentially eligible, examined for eligibility, confirmed eligible, included in the study, completing follow-up, and analysed | Please refer to the page 10 to 11, lines 237 to 243. |
|  |  | (b) Give reasons for non-participation at each stage | N/A |
|  |  | (c) Consider use of a flow diagram | N/A |
| Descriptive data | 14* | (a) Give characteristics of study participants (eg demographic, clinical, social) and information on exposures and potential confounders | Please refer to the page 10, lines 227 to 232. |
|  |  | (b) Indicate number of participants with missing data for each variable of interest | N/A |
| Outcome data | 15* | Report numbers of outcome events or summary measures | Please refer to the page 11 to 17, lines 244 to 297. |
| Main results | 16 | (*a*) Give unadjusted estimates and, if applicable, confounder-adjusted estimates and their precision (eg, 95% confidence interval). Make clear which confounders were adjusted for and why they were included | Please refer to the page 19 to 21, lines 298 to 335. |
|  |  | (*b*) Report category boundaries when continuous variables were categorized | Please refer to the page 10 to 17, lines 236 to 297. |
|  |  | (*c*) If relevant, consider translating estimates of relative risk into absolute risk for a meaningful time period | N/A |
| Other analyses | 17 | Report other analyses done—eg analyses of subgroups and interactions, and sensitivity analyses | N/A |
| Discussion | | |  |
| Key results | 18 | Summarise key results with reference to study objectives | Please refer to the page 22, lines 337 to 350. |
| Limitations | 19 | Discuss limitations of the study, taking into account sources of potential bias or imprecision. Discuss both direction and magnitude of any potential bias | Please refer to the page 25, lines 411 to 416. |
| Interpretation | 20 | Give a cautious overall interpretation of results considering objectives, limitations, multiplicity of analyses, results from similar studies, and other relevant evidence | Please refer to the page 22 to 25, lines 348 to 409. |
| Generalisability | 21 | Discuss the generalisability (external validity) of the study results | Please refer to the page 26, lines 424 to 435. |
| Other information | | |  |
| Funding | 22 | Give the source of funding and the role of the funders for the present study and, if applicable, for the original study on which the present article is based | Please refer to the page 27, lines 445 to 446. |

N/A= Not Available

*Give information separately for exposed and unexposed groups.

**Note:** An Explanation and Elaboration article discusses each checklist item and gives methodological background and published examples of transparent reporting. The STROBE checklist is best used in conjunction with this article (freely available on the Web sites of PLoS Medicine at [http://www.plosmedicine.org/,](http://www.plosmedicine.org/) Annals of Internal Medicine at [http://www.annals.org/,](http://www.annals.org/) and Epidemiology at [http://www.epidem.com/).](http://www.epidem.com/)) Information on the STROBE Initiative is available at [www.strobe-statement.org.](http://www.strobe-statement.org/)
