# Supplementary material for: Prevalence, trends and associated factors of hypertension and diabetes mellitus in Bangladesh: Evidence from BHDS 2011 and 2017–18
Source: PLoS One. 2022 May 3;17(5):e0267243. doi: 10.1371/journal.pone.0267243 (PMC9064112; doi:10.1371/journal.pone.0267243)
Supplement: S2 Table — (DOCX) [file pone.0267243.s002.docx]

**S2 Table. Test results of Variance Inflation Factor (VIF)**

Collinearity Diagnostics

| Variable | VIF | SQRT  VIF | Tolerance | R- Squared |
| --- | --- | --- | --- | --- |
| HTN | 1.21 | 1.10 | 0.8234 | 0.1766 |
| DM | 1.65 | 1.28 | 0.6069 | 0.3931 |
| HDC | 1.79 | 1.34 | 0.5599 | 0.4401 |
| Age | 1.17 | 1.08 | 0.8563 | 0.1437 |
| Gender | 1.22 | 1.10 | 0.8214 | 0.1786 |
| Educational level | 1.49 | 1.22 | 0.6718 | 0.3282 |
| Economic status | 1.54 | 1.24 | 0.6486 | 0.3514 |
| Body mass index (BMI) | 1.08 | 1.04 | 0.9285 | 0.0715 |
| Occupation type | 1.23 | 1.11 | 0.8141 | 0.1859 |
| Eating habit | 1.14 | 1.07 | 0.8746 | 0.1254 |
| Drinking coffee | 1.15 | 1.07 | 0.8707 | 0.1293 |
| Place of residence | 1.23 | 1.11 | 0.8101 | 0.1899 |
| Division | 1.01 | 1.01 | 0.9882 | 0.0118 |
| Mean VIF | 1.30 |  |  |  |

: HTN= Hypertension.

DM= Diabetes mellitus.

HDC= HTN-DM combined.

Variance Inflation Factor (VIF) was utilized to test the multicollinearity as we merged BDHS data: 2011 & 2017-18. The result is presented in S1 Table. Multicollinearity is considered to arise in the study if the value of VIF is 10 or above (or tolerances of .10 or less). The S1 Table reported that the existing study is free from multicollinearity problem.
