# Supplementary material for: Prevalence, trends and associated factors of hypertension and diabetes mellitus in Bangladesh: Evidence from BHDS 2011 and 2017–18
Source: PLoS One. 2022 May 3;17(5):e0267243. doi: 10.1371/journal.pone.0267243 (PMC9064112; doi:10.1371/journal.pone.0267243)
Supplement: S3 Table — (DOCX) [file pone.0267243.s003.docx]

**S3 Table.** **Baseline characteristics of the study participants aged 35 and over from Bangladesh Demographic and Health Survey (n=11,686)**

| Variables | n | % (95% CI) |
| --- | --- | --- |
| Outcome Variable |  |  |
| *HTN* |  |  |
| *No* | 8,319 | 71.19 (70.36-72.01) |
| *Yes* | 3,367 | 28.81 (27.99-29.64) |
| *DM* |  |  |
| *No* | 10,479 | 89.67 (89.11- 90.21) |
| *Yes* | 1,207 | 10.33 (9.79-10.89) |
| *HDC* |  |  |
| *No* | 11,223 | 96.05 (95.68-96.38) |
| *Yes* | 463 | 3.95 (3.62-04.32) |
| Explanatory Variable |  |  |
| *Age (in years)* |  |  |
| *35-39 years* | 1,975 | 16.90 (16.23-17.59) |
| *40-44 years* | 1,595 | 13.65 (13.04-14.29) |
| *45-49 years* | 1,577 | 13.49 (12.88-14.12) |
| *50-59 years* | 2,949 | 25.24 (24.46-26.04) |
| *60-69 years* | 2,039 | 17.45(16.77-18.15) |
| $\boldsymbol{\geq}$ *70 years* | 1,551 | 13.26 (12.66-13.89) |
| *Mean age in years,( mean*$\boldsymbol{\pm}$*SD)* | 11686 | 52.79 (12.99) |
| *Gender* |  |  |
| *Male* | 6,745 | 57.72 (56.82- 58.62) |
| *Female* | 4,941 | 42.28 (41.38-43.18) |
| *Educational level* |  |  |
| *No formal education* | 5,119 | 43.80 (42.90-44.70) |
| *Up to primary* | 3,429 | 29.34 (28.53-30.18) |
| *Up to secondary* | 2,141 | 18.32 (17.63- 19.04) |
| *Up to higher secondary* | 997 | 8.53 (8.04-9.05) |
| *Economic status* |  |  |
| *Poor* | 5,709 | 48.85 (47.95-49.76) |
| *Middle* | 2,412 | 20.64 (19.92-21.39) |
| *Rich* | 3,565 | 30.50 (29.68-31.34) |
| *Body mass index (BMI)* |  |  |
| *Normal (18.5-24.9)* | 6,614 | 56.60 (55.70- 57.49) |
| *Thin (<18.5)* | 2,801 | 23.97 (23.20-24.75) |
| *Overweight (>24.9)* | 2,271 | 19.44 (18.73-20.17) |
| *Occupation type* |  |  |
| *Physically inactive* | 7,031 | 60.16 (59.27-61.05) |
| *Physically active* | 4,655 | 39.84 (38.95-40.73) |
| *Eating habit* |  |  |
| *Specified* | 8,084 | 69.18 (68.34-70.01) |
| *Anything* | 3,602 | 30.82 (29.99-31.66) |
| *Drinking coffee* |  |  |
| *No* | 10,947 | 93.68 (93.22- 94.10) |
| *Yes* | 739 | 6.32 (5.90-6.78) |
| *Place of residence* |  |  |
| *Urban* | 2,776 | 23.76 (23-.24.54) |
| *Rural* | 8,910 | 76.24 (75.46-77) |
| *Division* |  |  |
| *Barisal* | 686 | 5.87 (5.46-6.31) |
| *Chittagong* | 1,924 | 16.46 (15.80-17.15) |
| *Dhaka* | 3,642 | 31.16 (30.33-32.01) |
| *Khulna* | 1,582 | 13.54 (12.93-14.17) |
| *Rajshahi* | 1,644 | 14.06 (13.45-14.71) |
| *Rangpur* | 1,514 | 12.95 (12.36-13.57) |
| *Sylhet* | 694 | 5.95 (5.53-6.39) |

CI= Confidence interval.

HTN= Hypertension.

DM= Diabetes mellitus.

HDC= HTN-DM combined
