# Supplementary material for: Prevalence, trends and associated factors of hypertension and diabetes mellitus in Bangladesh: Evidence from BHDS 2011 and 2017–18
Source: PLoS One. 2022 May 3;17(5):e0267243. doi: 10.1371/journal.pone.0267243 (PMC9064112; doi:10.1371/journal.pone.0267243)
Supplement: S4 Table — (DOCX) [file pone.0267243.s004.docx]

**S4 Table. Test results of Cochran-Armitage test**

| **Outcome** | ***P*^3^** |
| --- | --- |
| **HTN** | **<0.001** |
| **DM** | **<0.001** |
| **HDC** | **<0.001** |

HTN= Hypertension

DM= Diabetes mellitus

HDC= HTN-DM combined

P^3^**=** *P*-value for the Cochran–Armitage test
